# Preterm’s Nutrition from Hospital to Solid Foods: Are We Still Navigating by Sight?

**DOI:** 10.3390/nu12123646

**Published:** 2020-11-27

**Authors:** Beatrice Letizia Crippa, Daniela Morniroli, Maria Elisabetta Baldassarre, Alessandra Consales, Giulia Vizzari, Lorenzo Colombo, Fabio Mosca, Maria Lorella Giannì

**Affiliations:** 1 Fondazione IRCCS Cà Granda Ospedale Maggiore Policlinico, NICU, Via Commenda 12, 20122 Milan, Italy; daniela.morniroli@gmail.com (D.M.); alessandra.consales@gmail.com (A.C.); giulia.vizzari@unimi.it (G.V.); lorenzo.colombo@mangiagalli.it (L.C.); fabio.mosca@unimi.it (F.M.); maria.gianni@unimi.it (M.L.G.); 2 Department of Clinical Sciences and Community Health, University of Milan, Via Commenda 19, 20122 Milan, Italy; 3 Neonatology and Neonatal Intensive Care Unit, Department of Biomedical Science and Human Oncology, University of Bari “Aldo Moro”, 70124 Bari, Italy; mariaelisabetta.baldassarre@uniba.it

**Keywords:** preterm newborn, weaning, nutrition, post-discharge formula

## Abstract

As preterm birth rates are globally increasing, together with research on preterms’ peculiar needs, neonatologists are still facing the challenge of how to properly feed them. The need to strike a balance between excessive catch-up growth and extrauterine growth retardation, both leading to adverse outcomes, is made even more difficult by the broad range of preterms’ needs. Although mother’s fresh milk is undoubtedly the best nourishment, its availability during hospital stay is often lower than recommended, and its fortification at discharge is still an open issue. Formula milks are available as an alternative to breast milk. However, choosing the right formula requires a thorough evaluation of the infant’s perinatal history and targets. Last but not least, adequate timing and initiation of weaning in premature babies are still a poorly explored matter. This narrative review aims at evaluating the multitude of issues to consider when feeding preterms in the three stages of their first life: in-hospital care, discharge, and, eventually, weaning. Given the current absence of internationally shared guidelines, understanding the potential pitfalls of preterms’ nutrition could help us trace the right path for the right preterm.

## 1. Introduction

Preterm newborns account for 11% of live births in the world every day [1]. Prematurity is the leading cause of newborn mortality and morbidity in the first years of life. In fact, despite great advances in perinatal assistance and ongoing research, we are still not able to fully recreate the womb environment [2]. Preterm infants complete their organogenesis in a non-physiological environment. To increase the odds for survival in an adverse environment, neonates undergo structural and functional changes that lead to an increased risk of cardiovascular disease, arterial hypertension, and metabolic and renal disease [3]. Nutrition in the first 1000 days of life can play a pivotal role in this vulnerable population and especially in low birth weight (LBW) preterm babies. Reaching an optimal nutritional status, also through the provision of adequate amounts of essential macro- and micronutrients during critical periods in postnatal life, is necessary for physiologic brain development [4,5].

Appropriate early nutrition management is essential for preterm infants’ growth and development to prevent adverse health outcomes later in life and improve cognition in adulthood [6]. However, although evidence-based recommendations on LBW and extremely low birth weight (ELBW) infants in-hospital nutrition exist [6], little is known about the optimal nutritional approach after discharge and complementary feeding. Despite increasing evidence demonstrating that guidelines for term infants’ weaning are not applicable to the preterm population, to date, no specific recommendation for premature babies is universally accepted [5,7,8]. Differences in the development of motor skills and the digestive system between the preterm and term newborn limit the comparison between these two populations. Moreover, complementary feeding is a complex intervention [9], implemented in a critical time window, which exposes preterm babies to the risk of postnatal growth deviation (e.g., rapid catch-up growth or exacerbation of failure to thrive) [10].

The aim of the present narrative review is to provide an update on the available evidence on preterm nutrition, from the hospital setting to weaning.

## 2. Preterm Infants’ Postnatal Growth

Preterm infants’ growth can be classified into four distinct patterns [11]: (i) neonates adequate for gestational age (AGA) at birth without extrauterine growth retardation (EUGR); (ii) neonates AGA at birth but with EUGR; (iii) neonates small for gestational age (SGA) at birth without catch-up growth at discharge; (iv) neonates SGA at birth but with early catch-up growth at discharge. However, such a distinction is limited by the lack of consensus on EUGR definition: the cross-sectional definition consists of a weight below the 10th or 3rd centile (or another cut-off) at a given time, regardless of birth weight; conversely, the longitudinal definition refers to EUGR as a weight loss of more than 1 (or 2) Standard Deviations between birth and a given time. This discrepancy makes comparing the available studies and assessing nutritional interventions in clinical practice extremely difficult [12]. Fenton et al. [13] recently underlined the need to redefine the concept of EUGR and suggested stopping using the cross-sectional definition. They supported their ideas as follows: (i) cross-sectional EUGR is not predictive of adverse neurodevelopmental outcomes; (ii) it is based on weight only without considering head or length growth, body proportionality, body composition, or genetic potential; (iii) it ignores normal postnatal weight loss; (iv) it is based on an arbitrary statistical percentile cut-off [13]. Consistently, Peila et al. compared the two definitions and recommended the use of the longitudinal evaluation, which seems to better predict the auxological outcome at 24–30 months [12].

Preterms’ ideal growth patterns and standards are also a major topic of discussion in the scientific community. A preterm infant could not be considered a fetus in any sense (nutritional, metabolic, or physiologic), and the new INTERGROWTH-21st charts have recently changed the way we monitor preterms’ growth [14]. The authors suggested, as an appropriate comparator, a cohort of preterm newborns with uncomplicated intra- and extrauterine life. Currently, the INTERGROWTH-21st Preterm Postnatal Growth Standards can be used to assess preterm infants up to 64 weeks’ postmenstrual age, when they overlap with the WHO Child Growth Standards for term newborns [14].

The entity and the velocity of weight gain represent another controversial issue. Balancing poor growth and excessive catch-up is one of the greatest challenges in preterms’ nutrition. Early catch-up growth is very uncommon in clinical practice [15]. In fact, 80% of very low birth weight (VLBW) neonates with postnatal growth retardation and SGA infants show a slower catch-up growth within the first 2–3 years of life [15,16]. On the other hand, a rapid and early weight gain, especially in cases of low birth weight at birth, seems to predispose to adverse long-term effects (i.e., hypertension and cardiovascular risk, obesity, and type 2 diabetes). Although these effects are constitutively related to preterm birth, rapid catch-up growth seems to represent an additional risk factor [17]. Conversely, a poor growth rate in the first weeks of life correlates with reduced head circumference and, therefore, impaired neurodevelopment at one year [18].

## 3. In-Hospital Preterm Nutrition

Despite the increased awareness that postnatal growth of preterm babies cannot match the growth of healthy fetuses [14], EUGR represents a significant issue in preterm LBW infants, affecting 28, 34, and 16% of infants for weight, length, and head circumference, respectively [18]. At birth, the sudden interruption of placental support determines a nutritional crisis in the preterm infant, who can only count on poor caloric reserves. This deficit inversely correlates with the preterm’s birth weight. The administration of early enteral and parenteral support is the cornerstone of the nutritional management of preterm LBW and ELBW newborns [6,19]. A higher protein and energy intake during the first week after birth in ELBW neonates correlates with a faster head growth [20]; in turn, increased head circumference correlates with higher mental development index scores and lower risk of growth retardation [6,21,22]. Therefore, international evidence promotes the early initiation of fast and continuous enteral nutrition. Options for feeding preterm infants include mother’s expressed breast milk, donor expressed breast milk, fortified human milk (mother’s or donor), and formula milk. Human milk, preferably from the infant’s own mother, represents the best choice for preterm infants due to its many advantages, including protection from necrotizing enterocolitis and infections, and improved cognitive outcomes, particularly for VLBW infants, who experience the greatest benefits [23]. However, considering the increased macronutrient and micronutrient requirements of preterms, human milk alone, fed at the usual feeding volumes, may not provide sufficient nutrition, especially for VLBW infants [24]. This may result in slow growth rates, decreased fat-free mass gain, risk of neurocognitive impairment, specific deficiency states, such as osteopenia and zinc deficiency, and other poor health outcomes [24]. Human milk fortification is, therefore, needed to meet the preterm’s high requirements and has become part of preterm infants’ standard nutritional care in most Neonatal Intensive Care Units (NICUs). Standard fortification is effective and safe, but it does not always meet the high protein needs. For these reasons, the European Milk Bank Association (EMBA) Working Group on human milk encourages the use of “individualized fortification” to optimize nutrient intake [24]. Individualized fortification may be achieved through two different approaches: “adjustable fortification” or “targeted fortification”. The adjustable fortification method involves the periodic evaluation of blood urea nitrogen to adjust protein intake based on the infant’s actual metabolic response. It has the advantage of being practical and feasible in most NICUs while, at the same time, avoiding the risk of excessive protein intake. Conversely, the targeted fortification requires human milk analysis, thus allowing a tailored approach [24,25]. However, breast milk analysis is labor-intensive and expensive; thus, not all NICUs can afford this approach.

When sufficient maternal breast milk and donor human milk are not available, specific formula milks designed for preterm LBW infants can be used to meet their particular requirements [6]. The proper use of breast milk substitutes is based on adequate information and appropriate marketing and distribution, as well documented by WHO [26]. Preterm formula milks aim at providing nutrient intake to match intrauterine growth and are enriched in energy content, macronutrients, vitamins, minerals (i.e., iron and zinc), and trace elements [27]. Some formula milks available are enriched with long-chain polyunsaturated fatty acid (LCPUFA), particularly docosahexaenoic acid (DHA), necessary for the maturation of the brain and retina, and recommended by the European Society for Paediatric Gastroenterology Hepatology and Nutrition (ESPGHAN) [11]. Although preterm formula meets the nutrient needs of prematures, it fails to replicate the numerous immunological and bioactive factors contained in breast milk [28]. For this reason, breast milk represents the gold standard for every infant, and breastfeeding should always be promoted and protected when possible.

Based on current knowledge, one of the main challenges for the neonatologist is the definition of the preterm’s growth target in order to at least limit EUGR through an individualized nutritional approach (Table 1).

## 4. Post-Discharge Preterm Nutrition

At discharge, very preterm infants represent a nutritional challenge for the neonatologist. Two main nutritional issues at discharge are: choosing the right type of milk and continue fortifying mother’s milk. To make the best choice, many variables must be taken into consideration. Firstly, preterm infants tend to be discharged from the hospital earlier than the expected term. Overall, preterm infants may be sleepier at the time of discharge and may have more difficulties in latching, sucking, and swallowing than their full-term counterparts [15]. Additionally, the preterm population is extraordinarily diversified, encompassing babies with persistent morbidities (e.g., chronic lung disease, short bowel syndrome, etc.), high nutritional requirements and/or the need to limit the volume of feeds consumed, and babies with different body composition and growth patterns.

Consequently, an individualized approach is pivotal to optimize the post-discharge nutrition of premature infants. Different aspects and characteristics should be carefully assessed: the infant’s feeding skills, the presence of comorbidities that may require an increased caloric intake, the infant’s tolerance to feeding volumes, the need for weight recovery, the presence or absence of mother’s milk [26]. Breast milk represents the ideal nutrition for preterms [6] due to its well-known positive effects on cognitive skills and behavioral scores [29,34,35]. As mentioned above, in the NICU, some neonates need to receive fortified human milk to ensure adequate growth [29,36]. However, after discharge, the use of fortifiers of mother’s milk could be tricky: although nutritional requirements are still high, preterm babies’ feeding competency and their ability to suck on the breast are hopefully improved, and fortification could disrupt the routine of breastfeeding [29]. For this reason, the mother’s milk supplementation is often discontinued, with the subsequent risk of nutritional deficits and reduced weight gain during the first weeks after discharge [29,37]. Consequently, exclusive direct breastfeeding in preterm infants has been a matter of debate. Rozè et al. observed an association between exclusive breastfeeding at discharge and improved cognitive outcomes despite suboptimal initial weight gain in two independent cohorts of preterm infants (the Epidemiological Study on Small Gestational Ages, EPIPAGE, and the Loire Infant Follow-up Team, LIFT, cohorts) [29]. Their observation seems in contrast with previous studies documenting the association between suboptimal early postnatal nutrition and later cognitive dysfunction [38]. Accordingly, the authors introduced the interesting concept of the “apparent breastfeeding paradox”: the authors recognized that this “paradox” is probably only apparent because weight gain does not reflect body composition changes [29]. Specifically, many authors have described how human milk feeding could shape preterm infants’ body composition, increasing fat-free mass deposition in a dose-dependent manner [39,40]. The deposition of fat-free mass in preterm infants could play a role in preventing adverse neurodevelopment outcomes [2]. All these data are reassuring about exclusive breastfeeding after discharge, although the issue of the putative benefit of human milk supplementation after discharge remains open [29].

In a review focusing on human milk supplementation, Young et al. did not provide evidence that multinutrient fortification of breast milk for 3–4 months after hospital discharge affects growth rates during infancy or neurodevelopmental outcomes at 18 months corrected age [41]. However, several studies have shown some advantages of the use of human milk fortification after discharge [24], such as better lung function at six years [30], better anthropometric parameters in babies with a birth weight <1250 g up to one year of life [31], and better visual function [32]. Furthermore, in all these studies, human milk fortification was well tolerated, regardless of the mode of feeding, without any reported adverse effect on breastfeeding rates or gastrointestinal symptoms [24]. For these reasons, Arslanoglu et al. suggested considering human milk supplementation in breastfed babies who failed to adequately grow during hospitalization [24].

Although post-discharge nutrition is an everyday challenge in preterm care, to this day no shared recommendation is available for clinicians [36]. To the best of our knowledge, the last update on this topic can be found in the ESPGHAN Position Paper of 2006 [11]. According to ESPGHAN, premature infants without EUGR should continue exclusive breastfeeding whenever possible and, when formula-fed, they should receive standard infant formula with added LCPUFA. Conversely, infants with EUGR or at increased risk for long-term growth failure should receive fortified human milk to ensure adequate nutrient intake. In the case of formula feeding, special post-discharge formulas with high protein contents, calcium, phosphorus, zinc, and LCPUFA are suggested [11]. Although new reviews and metanalysis have been published [24,41,42,43], no evidence exists to support the use of the post-discharge formula. Besides, limited evidence suggests that feeding preterm infants with the preterm formula (generally available only for in-hospital use) after hospital discharge may increase growth rates up to 18 months post-term [42]. Similarly, Teller et al. found that nutrient-enriched diets after discharge have no negative effects, but, rather, they frequently improve growth parameters, especially in male infants [43]. According to ESPGHAN, special formula or human milk fortification could be continued up to at least 40 and possibly 52 weeks of gestational age [11]. Thereafter, no evidence, and therefore no clear indication, exists about how to feed preterm infants and when and how to wean them. In particular, it is important to gain further insight into specific nutritional post-discharge requirements needed for catch-up growth, balancing neurodevelopment and metabolic aspects (Table 1).

## 5. Nutrition during Weaning in Preterm Infants

Weaning represents a revolutionary period, both from a relational and developmental point of view. This is particularly true for preterm infants, considering their intrinsic immaturity and higher nutritional requirements. A recent survey, promoted by the Italian Society of Pediatrics and conducted among primary care pediatricians, showed the wide variability in the time of preterms’ introduction to complementary feeding and the type of foods proposed [10]. The European Food Safety Authority (EFSA) Panel on Nutrition recently underlined the lack of evidence [44], reporting a single randomized controlled trial (RCT) that concluded that weaning at 4 vs. 6 months corrected age does not affect anthropometric measures at 12 months corrected age in preterm infants <34 weeks of gestation [33]. However, the Panel raised doubts on the generalizability of these results to the European setting, given the specific characteristics of the population considered by Gupta et al.’s RCT. Anyways, evidence based on observational studies may direct optimal nutritional management during this critical period. For instance, a prospective cohort study by Spiegler et al. found no effect on height and weight at 2 years of age of the early introduction of complementary foods [45]. Similarly, Morgan et al. performed a pooled analysis of prospective studies that led to the conclusion that weaning before or after 12 weeks post-term hardly influences health outcomes up to 18 months [46]. Finally, the retrospective case-control study by Yrjänä et al. concluded that the early introduction of semisolid foods does not influence the incidence of food allergy or atopic dermatitis among preterm infants, suggesting that their gut-associated lymphoid tissue is ready for complementary foods within 3–6 months of birth, regardless of gestational age [47]. In conclusion, it appears that the optimal age for the introduction of complementary food for preterm infants remains a controversial issue, and more RCTs are needed to confirm the results obtained so far.

Transition to complementary food is often an uneasy path. Preterm’s eating difficulties may be related to their intrinsic immaturity, neurological deficits, and co-morbidities, or even have psychological roots caused by the multiple and unpleasant procedures undergone during hospitalization (e.g., tube feeding, intubation, etc.). Moreover, the involvement of emotional factors could play a significant role and need to be considered, especially in growth-restricted babies, whose growth rate is often a cause for concern for parents [48,49]. We can recognize two critical aspects, and we could hypothesize possible practical approaches. If the acceptance of semi-solid food is still incomplete or problematic, attention should be paid to the intake of micronutrients. For this reason, in our context, premature infants are discharged with iron and multivitamin supplementation. Secondly, if catch-up growth has not been reached by the time of weaning, it is important to guarantee a high protein and caloric intake by choosing the right formula milk or individualizing foods to propose (Table 1). The choice of the type of formula milk (i.e., post-discharge or standard formula) is also strictly dependent on the milk volume the baby can consume, although some preterm infants can regulate their volume intake to compensate for low nutrient content. Less mature preterms or infants with comorbidities often have feeding tolerance issues due to their immature feeding skills but higher energy requirements [5].

To date, no joint indication exists on which type of formula milk should be given during complementary feeding if breast milk is not available.

## 6. Conclusions

The epigenetic effect of nutrition on general health has been already established. This effect is even more clear for premature infants, whose nutrition, complicated by clinical conditions, is a critical aspect of their forced extrauterine development. To this day, no agreement has been reached to design a shared path for clinicians, but a possible trace could be given by personalized nutrition. At discharge, the authors believe that the following aspects need to be considered when introducing complementary foods: (i) growth target; (ii) nutritional requirements; (iii) feeding skills. In fact, just as in the upcoming years, healthcare and medications will become increasingly more oriented to the single patient, individualized nutrition based on premature infant’s key characteristics could be the only way to guarantee this vulnerable population the best start in life.

## Figures and Tables

**Table 1:** Summary of recent evidence and challenges in preterms’ nutrition.

	Recent Evidence	Challenges
In-hospital nutrition	Preterm Postnatal Growth Standards [14] “Targeted” human milk fortification [24]	Avoid/limit extrauterine growth retardation Individualize the nutritional approach Define the growth target
Post-discharge nutrition	Preterm Postnatal Growth Standards [14] Breastfeeding paradox [29] Advantages of the use of human milk fortification after discharge [30,31,32]	Define entity and velocity of catch-up growth, balancing neurodevelopmental and metabolic aspects Individualize the nutritional approach Define the nutritional requirements Consider feeding skills and emotional factors
Nutrition during weaning	Preterm Postnatal Growth Standards up to 64 weeks’ postmenstrual age [14] Weaning between 4 and 6 months corrected age [33]	Avoid the risk of postnatal growth deviation (e.g., rapid catch-up growth or exacerbation of failure to thrive) Individualize the nutritional approach Consider feeding skills and emotional factors Consider acceptance of semi-solid food and micronutrient intake
